# Emergence of *RAS* mutations in patients with metastatic colorectal cancer receiving cetuximab-based treatment: a study protocol

**DOI:** 10.1186/s12885-019-5826-7

**Published:** 2019-06-28

**Authors:** Shang-Hung Chen, Hsiang-Lin Tsai, Jeng-Kai Jiang, Yung-Chuan Sung, Ching-Wen Huang, Yu-Min Yeh, Li-Tzong Chen, Jaw-Yuan Wang

**Affiliations:** 1 National Institute of Cancer Research, National Health Research Institutes, Tainan, Taiwan; 2 Division of Hematology and Oncology, Department of Internal Medicine, National Cheng Kung University Hospital, College of Medicine, National Cheng Kung University, Tainan, Taiwan; 3 Division of Colorectal Surgery, Department of Surgery, Kaohsiung Medical University Hospital, Kaohsiung Medical University, Kaohsiung, Taiwan; 4 Department of Surgery, Faculty of Medicine, College of Medicine, Kaohsiung Medical University, Kaohsiung, Taiwan; 5 Division of Colon & Rectal Surgery, Department of Surgery, Taipei Veterans General Hospital Medical School, National Yang-Ming University, Taipei, Taiwan; 6 School of Medicine, Fu-Jen Catholic University, New Taipei, Taiwan; 7 Division of Hematology/Oncology, Internal Medicine, Cathay General Hospital, Taipei, Taiwan; 8 Graduate Institute of Clinical Medicine, National Cheng Kung University Hospital, College of Medicine, National Cheng Kung University, Tainan, Taiwan; 9 Department of Internal Medicine, Kaohsiung Medical University Hospital, Kaohsiung Medical University, Kaohsiung, Taiwan; 10 Graduate Institute of Clinical Medicine, College of Medicine, Kaohsiung Medical University, Kaohsiung, Taiwan; 11 Graduate Institute of Medicine, College of Medicine, Kaohsiung Medical University, Kaohsiung, Taiwan; 12 Center for Cancer Research, Kaohsiung Medical University, Kaohsiung, Taiwan

**Keywords:** *RAS* mutation, Metastatic colorectal cancer, Cetuximab, Liquid biopsy

## Abstract

**Background:**

In the management of patients with *RAS* wild-type metastatic colorectal cancer (mCRC), anti-epidermal growth factor receptor (EGFR) therapies have demonstrated a clinical benefit, with longer survival. However, the correlation between the emergence of circulating *RAS* mutations and secondary resistance to anti-EGFR therapies requires further elucidation. In this study, we aim to examine evolutionary changes in *RAS* mutations through liquid biopsy in patients with mCRC during and after anti-EGFR therapy.

**Methods:**

A total of 120 patients diagnosed with *RAS* wild-type mCRC will be enrolled in this study. Patients will receive a cetuximab-based infusional 5-fluorouracil regimen as first-line treatment. Cetuximab-based treatment is expected to continue until disease progression, intolerable toxic effects, or withdrawal of consent. Blood samples from enrolled patients will be collected before and then every 3 months during cetuximab-based treatment and also at disease progression. These blood samples will be evaluated for *RAS* resistance mutations by using the MassARRAY platform. The primary endpoint is the percentage of *RAS* mutations detected in circulating DNA from patients during cetuximab treatment. The correlation between the tumor response and survival outcomes of these patients and the emergence of circulating *RAS* mutations will be further analyzed.

**Discussion:**

Liquid biopsy is a powerful technology that can represent tumor heterogeneity in a relatively noninvasive manner. Because *RAS* mutations play a major role in resistance to anti-EGFR therapy for mCRC, examining evolutionary changes in these mutations during such treatment through liquid biopsy would be useful. After comprehensively analyzing the emergence of circulating *RAS* mutations and its clinical relevance in this study, our results should provide practical guidance on anti-EGFR therapy for mCRC.

**Trial registration:**

The date of trial registration (NCT03401957) in this study was January 17, 2018.

**Electronic supplementary material:**

The online version of this article (10.1186/s12885-019-5826-7) contains supplementary material, which is available to authorized users.

## Background

Colorectal cancer (CRC), a neoplasm arising from the large bowel, is a common and lethal disease with approximately 1,100,000 new cases and 550,000 deaths worldwide in 2018 [1]. In Taiwan, CRC is the most commonly diagnosed cancer (15,579 new cases in 2015) and the third most common cause of cancer-related deaths (5687 deaths in 2015) [2]. Nearly 20% of newly diagnosed cases of CRC are metastatic at initial presentation; a certain proportion of patients in early stages would also develop metastases even after curative surgery [3]. Systemic treatment is generally recommended for metastatic CRC (mCRC). In addition to conventional chemotherapy drugs, several agents targeting the molecular drivers of CRC pathogenesis, including signaling pathways mediated by the epidermal growth factor receptor (EGFR) and vascular endothelial growth factor, have been applied in such patients, with increasing survival rates [4–8].

Cetuximab is an EGFR-targeted monoclonal antibody with established clinical benefits as a component of first-line treatment for patients with *RAS* wild-type mCRC [7, 8]. The predictive role of *RAS* mutations in the clinical responses of mCRC to anti-EGFR therapies has been demonstrated in several pivotal studies [7–11]. RAS belongs to a family of small G proteins, including HRAS, KRAS, and NRAS, which are responsible for ligand-dependent receptor activation. In general, *KRAS* mutations are found in approximately 40% of patients with CRC, *NRAS* mutations are about 3%, and *HRAS* mutations are relatively rare [11, 12]. Mutations at key sites within the *RAS* family cause constitutive activation of RAS-associated signaling, rendering anti-EGFR therapies ineffective for mCRC. Therefore, the identification of *RAS* mutations in tumor tissues to determine patients that are more likely to benefit from anti-EGFR therapies has become standard in the pretreatment management of patients with mCRC [12]. Moreover, acquired resistance inevitably appears in some patients after the initial response to cetuximab, thus limiting the clinical benefit of this anti-EGFR antibody. The emergence of *RAS* mutations is also potentially responsible for acquired resistance to cetuximab in patients with mCRC [13–15]. *RAS* mutations have been identified after anti-EGFR therapies in approximately 50% of patients with *RAS* wild-type mCRC [13, 14]. In addition, genetic alterations in *BRAF*, a downstream effector of the EGFR signaling pathway, have been found in around 5% of patients with CRC. Some meta-analyses have shown that *BRAF*-mutant CRCs are associated with the low clinical efficacy of anti-EGFR therapies [11, 12]. Accordingly, dynamic monitoring for the emergence of activation mutations of effectors downstream located in EGFR signaling pathway, especially *RAS* mutations in patients undergoing anti-EGFR therapies can be a useful tool to determine tumor response and ongoing patient care.

During cancer progression, circulating nucleic acids carrying specific genetic alterations of tumor cells (circulating tumor DNA, or ctDNA) from both primary and metastatic sites can enter the bloodstream [16]. Liquid biopsy is a newly developed technique capable of detecting these genetic alterations, especially specific base nucleotide substitutions from ctDNA, through blood sampling. Certain point mutations of tumor cells from circulating free DNA (cfDNA) in the plasma of patients with a given cancer type, including CRC, have been identified [17, 18]. An analysis of cfDNA through liquid biopsy avoids the limitations of tumor tissue-based mutation analysis. Therefore, this minimally invasive technique can offer the advantage of continuously monitoring the major genotype represented in tumor cells with a complex heterogeneity. Nevertheless, substantial challenges remain because ctDNA often represents only a small fraction of total cfDNA [19–21]. Standard sequencing approaches, such as the Sanger sequencing method, can only detect tumor-specific mutations in patients with a heavy tumor burden. A personalized approach using sensitive detection tools, such as next generation sequencing (NGS), is also not feasible in routine clinical practice due to the high cost and demand for qualified research personnel.

More recently, a mass spectrometry-based technique combined with a single-base extension polymerase chain reaction (PCR) has been used to investigate genotyping across a variety of human cancers [22, 23]. This high-throughput technique, called the MassARRAY platform (Sequenom, Brisbane, Australia), can access up to 40 single-nucleotide polymorphisms in a single reaction with satisfactory sensitivity and specificity. Results from other studies have verified the concordance of genotyping in CRC patient-matched plasma and tumor tissue samples using this mass spectrometry platform [23, 24]. Therefore, in this prospective study, we will use this platform to examine *RAS* mutations in serial blood samples collected from patients with mCRC undergoing cetuximab treatment. To determine a more effective anti-EGFR therapeutic strategy for mCRC, the correlation between the clinical response of tumors to cetuximab and the emergence of resistant mutations will also be analyzed.

## Methods/design

### Study design

**Fig. 1:**
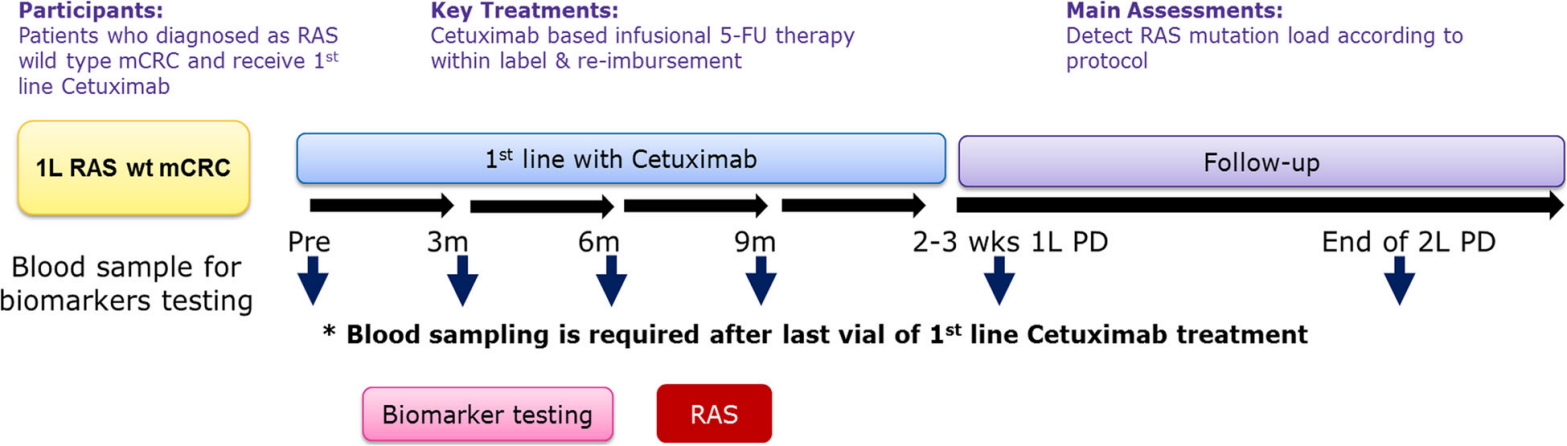
Schematic flowchart providing an overview of the study design; wt, wild-type; mCRC, metastatic colorectal cancer; 1 L, first-line treatment; 2 L, second-line treatment; PD, progression of disease

This single-arm, non-interventional, uncontrolled, multicenter study will evaluate the emergence of *RAS* mutations in patients with mCRC receiving a cetuximab-based regimen as first-line treatment. In this investigator-initiated study, patients diagnosed with *RAS* wild-type mCRC will be recruited. Patients for whom treatment is planned with a cetuximab-based regimen under the locally approved label will be enrolled. In addition to cetuximab, infusional 5-fluorouracil is required and combination with oxaliplatin or irinotecan is allowed in first-line treatment. On the basis of the best scientific knowledge, clinical practice for each patient is determined entirely by the responsible investigator. This cetuximab-based treatment is expected to continue until disease progression, intolerable toxic effects, or withdrawal of consent. Blood samples from patients enrolled in this study will be collected before the start of cetuximab-based chemotherapy and then every 3 months during first-line treatment. Blood sampling is also required within 3 weeks of disease progression following cetuximab and second-line treatments. The blood samples will be sent to the central laboratory at Taipei Institute of Pathology and evaluated for the *RAS* genotype by using the MassARRAY technique. Pretreatment tissue sections will also be re-evaluated for the *RAS* genotype by using this technique if a discordance of *RAS* genotype occurs between the tissue and blood samples of the same patient. This study is briefly outlined in Fig. 1.

### Study objectives

The primary endpoint is the percentage of *RAS* mutations detected in the cfDNA of patients with mCRC during first-line cetuximab treatment. The secondary objectives are the following endpoints: 1. duration between the start of cetuximab treatment and the new detection of a *RAS* mutation; 2. percentage of mutated alleles detected at disease progression; 3. clinical responses and metastasis resection rates during first-line cetuximab treatment; 4. progression-free and overall survival of patients receiving first-line cetuximab treatment; 5. correlations between *RAS* resistance mutations after cetuximab treatment (occurrence and levels) and clinical survival outcomes; 6. total cetuximab dosage in first-line treatment; and 7. correlations between irinotecan or oxaliplatin dosage and acquired resistance to cetuximab.

### Eligibility criteria

In this multicenter study, we intend to enroll 120 patients with wild-type *RAS* mCRC from four participating hospitals, namely Kaohsiung Medical University Hospital, Taipei Veterans General Hospital, Cathay General Hospital, and National Cheng Kung University Hospital. The selection of patients is at the discretion of the investigator. Inclusion and exclusion criteria are listed below.

#### Inclusion criteria

1. Patients with mCRC for whom a cetuximab-based regimen is planned as first-line treatment after considering routine clinical practice, the locally approved label, and the best scientific knowledge. The choice of the chemotherapy regimen for first-line treatment is also at the sole discretion of the investigator, based upon routine clinical practice.
2. Patients aged 20 years and above.
3. Patients pathologically and molecularly diagnosed with wild-type *RAS* CRC.
4. Patients willing to provide blood samples during the study.
5. Patients willing and able to provide signed informed consent.

#### Exclusion criteria

1. Patients with a history of any anti-EGFR therapy.
2. Contraindications to cetuximab as per the locally approved label.

### Blood sampling

Blood will be obtained from an arterial or venous line according to standard phlebotomy technique, with a Cell Free DNA collection tube (Roche) used for sampling. Blood specimens will be shipped at room temperature within 24 h, and plasma preparation and cfDNA extraction will be performed within 7 days of sampling at Taipei Institute of Pathology.

### *RAS* mutation analysis of cfDNA and tissue samples

Before mutation analysis, cfDNA will be extracted using the Cobas cfDNA sample preparation kit, and a representative tumor sample (primary or metastasis) will be made available for a repeat *RAS* mutation analysis in case of any discordance between the tumor tissue and cfDNA analyses. Three to five sections of a formalin-fixed paraffin-embedded (FFPE) tissue for DNA extraction will be made using the QIAamp DNA FFPE tissue kit. Mutations will be detected using a MassARRAY platform combined with the single allele base extension reaction (SABER) technique (Agena, San Diego, California, USA). In a SABER reaction using the iPLEX enzyme, SABER terminator mix, and extension primer mix (iPLEX Pro kit, Agena), clinically relevant mutations in *KRAS, NRAS* and *BRAF* genes are targeted (Additional file 1: Table S1). After the addition of a cation exchange resin to remove residual salt from the reactions, a purified primer extension reaction is loaded onto the matrix pad of a SpectroCHIP bioarray (Sequenom) by using a MassARRAY nano-dispenser and assayed on the MassARRAY platform. The mutation peaks are identified as a higher than background intensity compared with a non-mutation sample pool.

### Schedule of assessments

Patients will undergo *RAS* mutation analysis of cfDNA every 3 months during cetuximab-based first-line treatment and within 3 weeks of disease progression after first- and second-line treatments. During the study period, the assessment of patients will be scheduled according to the clinical judgment of the responsible investigator. The tumor response will likewise be assessed from the imaging scan at the investigator’s judgment.

### Sample size calculation

The present study aims to identify the frequency of *RAS* mutations in cfDNA during cetuximab treatment. In earlier studies, 21 and 33% of patients with *RAS* wild-type CRC at baseline displayed *KRAS* mutations at week 24 and 26, respectively [13–15]. Sample size is based on test power considerations by using a confidence limit. When the Wilson score method was used, a sample size of 110 produced two-sided 95% confidence intervals (CIs) of 0.169, 0.176, and 0.180 when the sample proportion was 0.300, 0.350, and 0.400, respectively [25]. Taking a 10% dropout rate into account, 120 patients should be enrolled in this study.

### Statistical analysis

The National Health Research Institutes is responsible for data management and statistical analysis. In general, descriptive statistics are used in this study. All patients receiving at least 8 weeks of treatment and having at least one post-baseline *RAS* mutation in their cfDNA will be eligible for clinical efficacy and outcome evaluation (efficacy population). The frequency of *RAS* mutations will be calculated and presented as a number, percentage, and 95% CI for the efficacy population. Clinical responses to cetuximab treatment will likewise be presented as a frequency, percentage, and 95% CI. Cox proportional hazard models will be used to investigate the effect of *RAS* mutations on time-to-event endpoints, including progression-free survival (PFS) and overall survival (OS). Other major clinical variables will also be included in these Cox models (e.g., disease characteristics or type of chemotherapy). Kaplan–Meier survival curves will be produced for PFS and OS of patients with and without genetic mutations. Kaplan–Meier methods will also be applied to the onset time of newly detected *RAS* mutations in cfDNA. The onset time of *RAS* mutations will be censored in patients without mutations at the last available measurement of cfDNA.

### Ethical considerations

The final protocol of this study was approved by the ethics committee of the National Health Research Institutes, with reference number EC1060904. Official approval has also been obtained from the ethics committee of Kaohsiung Medical University Hospital (reference number: KMUHIRB-GII-20170027), Taipei Veterans General Hospital (reference number: 2017–12-003A), Cathay General Hospital (reference number: CGH-P107013), and National Cheng Kung University Hospital (reference number: A-BR-106-045). The study has been registered on the ClinicalTrial.gov website, with identification number NCT03401957. The trial will be performed in accordance with the 7th version of the World Medical Association’s Declaration of Helsinki, the International Council for Harmonization’s E6 (R2) Guideline for Good Clinical Practice, and regulatory laws in Taiwan. Prior to participation in this study, written informed consent will be obtained from each patient.

## Discussion

The release of ctDNA into the bloodstream can originate from apoptosis, necrosis, and probably also the active secretion of tumor cells [16, 26]. Although ctDNA would only account for a certain portion of cfDNA in patients with cancer, circulating nucleic acids from tumor cells could be detected through the development of molecular quantification techniques such as NGS and digital PCR (dPCR) [17, 27]. Over the past few years, clinical applications of liquid biopsy, which examines existing genetic alterations through ctDNA, have been widely explored for early diagnosis, recurrence/metastasis monitoring, and prognostic value in patients with a variety of cancers [19, 28, 29]. Apart from such applications, which are similar to conventional tumor markers, another potential advantage of ctDNA is the ability to detect specific genetic variations known to cause resistance to anticancer treatments, especially targeted therapies. This would enable monitoring tumor response and even modifying early treatment in patients during targeted anticancer therapy. The clinical benefits of anti-EGFR therapies have been demonstrated in patients with mCRC; however, mutations in *RAS* are reportedly linked to primary anti-EGFR therapy resistance [7–11]. Initial retrospective analyses of pivotal studies have shown that activating mutations in *KRAS* exon 2 are predictive of poor response to anti-EGFR antibodies. Moreover, recent post hoc researches from clinical studies have shown that mutations outside of those in *KRAS* exon 2, including exons 3 and 4 of *KRAS* and exons 2, 3 and 4 of *NRAS,* also own the predictive value of a low response to anti-EGFR antibodies [11, 12]. Apart from *RAS* mutations, some studies have demonstrated that genomic alterations in other effectors of EGFR pathway, such as *BRAF* mutations, can be negative predictive biomarkers for anti-EGFR therapies [11, 12]. Therefore, the research aimed at monitoring the emergence of genomic alterations in effectors of EGFR pathway and elucidating their connection with acquired resistance to anti-EGFR therapies in patients with mCRC is warranted.

Several studies have reported a correlation between the emergence of circulating *RAS* mutations and the acquisition of resistance to anti-EGFR therapies in patients with mCRC [13, 14, 30, 31]. The potential value of these studies for clinical application is summarized in Table 1. Among patients with *RAS* wild-type mCRC, the detection rate of *RAS* mutations in ctDNA was 13–60% when secondary resistance to anti-EGFR therapies is ensured. In a cornerstone study by Misale et al., the onset of *KRAS* mutations in ctDNA analysis could be detected as early as 10 months prior to disease progression through radiological documentation [14]. However, in another pioneering study by Diaz et al., circulating *KRAS* mutations generally occurred 5–6 months after anti-EGFR therapy [13]. Overall, the detectable quantity of mutant ctDNA increases gradually during the development of secondary resistance. Nevertheless, a sudden increase in circulating *KRAS* mutations would be a warning sign. As reported by Toledo et al., fulminant tumor progression clinically follows an abrupt increase in mutant ctDNA [30].

**Table 1:** Summary of studies monitoring *RAS* mutations in ctDNA of mCRC patients receiving anti-EGFR therapies

Reference	Methods for ctDNA measurement	Patients (n)	Previous chemotherapy exposure	Anti-EGFR therapy	Rate of detected circulating *RAS* mutations during treatment (%)	Potential clinical value
Diaz LA et al. [13]	BEAMing	28	yes	panitumumab	38	Circulating *KRAS* mutations generally occurred 5 to 6 months after anti-EGFR therapy.
Misale S et al. [14]	Pyrosequencing or BEAMing	10	yes	cetuximab or panitumumab	60	Circulating *KRAS* mutations were frequently detected in acquired resistance to anti-EGFR therapies.
Toledo RA et al. [30]	BEAMing	23	no	cetuximab	13	Abrupt increase in mutant cfDNA correlated with eminent clinical deterioration.
Vidal J et al. [31]	BEAMing	18	unknown	cetuximab or panitumumab	39	Circulating *RAS* mutations correlated with acquired resistance to anti-EGFR therapies.

Although a significant association between the emergence of circulating *RAS* mutations and secondary resistance to anti-EGFR therapies has been revealed by these studies [13, 14, 30, 31], the relatively small number of enrolled patients limits their potential value for clinical application. The retrospective nature of these studies also hinders confidence in the utility of liquid biopsy in monitoring anti-EGFR therapy response. Large prospective clinical studies are necessary to elucidate the clinical relevance of using ctDNA to dynamically monitor resistant genetic variants during anti-EGFR therapies. Therefore, we designed this prospective study to investigate the emergence of *RAS* mutations in the ctDNA of patients with *RAS* wild-type mCRC receiving anti-EGFR therapy as first-line treatment. To the best of our knowledge, this study, which intends to recruit 120 patients, has the largest patient enrollment among the ongoing trials of the efficacy of liquid biopsy in mCRC. To further explore the clinical relevance of liquid biopsy, tumor response to anti-EGFR therapies and survival outcomes will be statistically compared with serial alterations of circulating *RAS* mutations in these patients. The development of drug resistance within tumor cells is believed to be a dynamic process of ecological evolution [32]. Consecutive changes in mutant ctDNA after the discontinuation of anti-EGFR therapies still remain unclear. This study will analyze the mutant ctDNA of patients with mCRC not only during anti-EGFR therapy as first-line treatment but also at disease progression after second-line treatment. Rechallenge with a previously administered cetuximab-based regimen has been reported to alleviate tumor progression in patients with refractory mCRC [33]. Evolutionary changes of the mutation burden in ctDNA revealed by this current study can offer a fundamental rationale for an anti-EGFR therapy rechallenge strategy.

Two main techniques for detecting genetic variations in ctDNA are currently in use. The first incorporates a nontargeted method using genome-wide analysis of ctDNA, such as an NGS-based technique. In a recent retrospective analysis from the ASPECCT study, Peeters et al. have reported that higher mutant allele frequencies in EGFR pathway genes detected in cfDNA, using an NGS-based technique, correlate with poor outcome of patients with mCRC receiving anti-EGFR antibodies [18]. Although this approach can provide plentiful genetic information, its lower cost- effectiveness would limit its utilization in clinical practice, especially when distinct genetic variants are to be tested. The second technique involves a targeted approach including the analysis of known genetic variants, such as a dPCR-based technique. This approach offers more sensitive detection of the specific mutant ctDNA that is the focus of research. However, a lack of high-throughput analysis is one major drawback of such dPCR-based techniques. Recently, the MassARRAY platform, an approach integrating a mass spectrometry-based technique with single-base extension PCR, has been developed for liquid biopsy applications. This integrated technique could provide the advantage of high-throughput detection of multiplex genetic variations [24]. The sensitivity of mutant ctDNA detection in this technique was comparable to that of dPCR-based techniques in patients with CRC [34]. Therefore, the MassARRAY platform is employed in this study to analyze *RAS* mutations in the ctDNA of patients during and after anti-EGFR therapy.

In summary, this prospective study is designed to investigate the emergence of *RAS* mutations in the ctDNA of patients with *RAS* wild-type mCRC during a cetuximab-based regimen as first-line treatment and at disease progression after cetuximab and second-line treatments. To determine potential clinical applications, dynamic alterations of circulating *RAS* mutations will be correlated with the clinical outcomes of these patients. The results of this study will offer substantial, valuable information for an anti-EGFR therapeutic strategy in patients with mCRC.

## Additional file

Additional file 1: **Table S1.** The list of activation mutations detected using the MassARRAY system. (DOCX 17 kb)

## Data Availability

Not applicable.

## Abbreviations

cfDNA: Circulating free DNA
CI: Confidence interval
CRC: Colorectal cancer
ctDNA: Circulating tumor DNA
dPCR: Digital PCR
EGFR: Epidermal growth factor receptor
FFPE: Formalin-fixed paraffin-embedded
mCRC: Metastatic colorectal cancer
NGS: Next generation sequencing
OS: Overall survival
PCR: Polymerase chain reaction
PFS: Progression-free survival
SABER: Single allele base extension reaction
