# Supplementary material for: Emergence of RAS mutations in patients with metastatic colorectal cancer receiving cetuximab-based treatment: a study protocol
Source: BMC Cancer. 2019 Jun 28;19:640. doi: 10.1186/s12885-019-5826-7 (PMC6599252; doi:10.1186/s12885-019-5826-7)
Supplement: Supplementary file 1 — Table S1. The list of activation mutations detected using the MassARRAY system. (DOCX 17 kb) [file 12885_2019_5826_MOESM1_ESM.docx]

Additional table 1. The list of activation mutations detected using the MassARRAY system.

| **Gene** | **Exon** | **Codon** | **Mutation** |
| --- | --- | --- | --- |
| *KRAS* | 2 | 12 | p.G12C |
|  |  |  | p.G12R |
|  |  |  | p.G12S |
|  |  |  | p.G12A |
|  |  |  | p.G12D |
|  |  |  | p.G12V |
|  |  | 13 | p.G13C |
|  |  |  | p.G13R |
|  |  |  | p.G13S |
|  |  |  | p.G13A |
|  |  |  | p.G13D |
|  |  |  | p.G13V |
|  | 3 | 59 | p.A59T |
|  |  |  | p.A59G |
|  |  | 61 | p.Q61K |
|  |  |  | p.Q61E |
|  |  |  | p.Q61P |
|  |  |  | p.Q61L |
|  |  |  | p.Q61R |
|  |  |  | p.Q61H |
|  | 4 | 117 | p.K117N |
|  |  | 146 | p.A146P |
|  |  |  | p.A146T |
|  |  |  | p.A146V |
| *NRAS* | 2 | 12 | p.G12C |
|  |  |  | p.G12R |
|  |  |  | p.G12S |
|  |  |  | p.G12A |
|  |  |  | p.G12D |
|  |  |  | p.G12V |
|  |  | 13 | p.G13C |
|  |  |  | p.G13R |
|  |  |  | p.G13S |
|  |  |  | p.G13A |
|  |  |  | p.G13D |
|  |  |  | p.G13V |
|  | 3 | 59 | p.A59T |
|  |  |  | p.A59G |
|  |  | 61 | p.Q61K |
|  |  |  | p.Q61E |
|  |  |  | p.Q61P |
|  |  |  | p.Q61L |
|  |  |  | p.Q61R |
|  |  |  | p.Q61H |
|  | 4 | 117 | p.K117N |
|  |  | 146 | p.A146P |
|  |  |  | p.A146T |
|  |  |  | p.A146V |
| *BRAF* | 15 | 600 | p.V600E |
